# On External Use of Chloroform

**Published:** 1848-09

**Authors:** 


*On external use of Chloroform.*—In the addenda of Braithwaite's Retrospect part 17th, we find the following account of the observations of Mr. Nunneley on the external use of chloroform.—Ed. Buff. Med. Jour.

In concluding our remarks on the use of anæsthetic agents in medicine, we cannot help adverting to some very important and novel views on this subject, brought before the branch meeting of the Provincial Association at Leeds, on June 17th, 1848, by Mr. Nunneley of that town. This gentleman stated that for many months he had been engaged in making experimental researches on those agents, with a view to ascertain, as far as possible, the modus operandi, the doses which may be borne with impunity, and the different modes of application: as well as, in cases of an overdose, the best means to be adopted to counteract it.

His experiments have not merely extended to the common anæsthetic agents employed, such as ether and chloroform, but he has been endeavoring to ascertain whether or not there may be some others, which may either be more safely administered, or may possess still greater advantages than the usual agents employed. He stated that he believed it not improbable that it would ultimately be found that all those preparations, which have a *radical basis*, (in the language of modern chemistry,) such as acetic ether, bisulphuret of caibon, aldehyde, and many others of an analogous character, upon some of which he had made extensive experiments, would be found to possess similar properties on the animal economy. He did not intend to enter on this important question generally, which he reserved for another occasion.

This much Mr. Nunneley was prepared to state, *that chloroform appeared to be the most deleterious to life*, to require the greatest care in administration, and that the boundary up to a fatal dose is by no means well-marked—that of two animals, in apparently the same condition, the same dose being given in precisely the same way to both, the one will speedily die, while the other will bear it with impunity,—that from the effects observed, he has reason to think the ultimate effects are in some respects not dissimilar to those produced by prussic acid,—that to some animals, as for instance the newt, the frog, the toad, some fish, slugs, snails, and some insects, the effects are *more* rapidly fatal than prussic acid of Scheele's strength; and that even in higher animals, when under the influence of an incomplete dose, or recovering from the effects of a large dose of either chloroform or prussic acid, the phenomena are in many respects very similar; and further, that the numerous post mortem examinations which he has made, fully corroborated this opinion. He stated that acetic ether, with which he had made numerous experiments, possessed very considerable anæsthetic powers,—that bisulphuret of carbon also possesses to some extent similar power, and so far as his experiments go, it is very important to add, that this power is of a safe character, the animal speedily recovering.

But of all these remedies he believes that sulphuric ether will be the safest and least noxious to life. On these points Mr. Nunneley intends hereafter to lay his experiments, already very numerous and varied, before the profession. His chief object on the present occasion was to call the attention of the profession to experiments proving, as he thinks, the value and safety of a *new mode* of administering these agents.

His object on the present occasion was to show that the action of all, or most of these agents, might be produced *locally by local application*, the sensorium being unaffected, consciousness being retained, and the limbs not subjected to their influence, being unaffected. He stated that either by *immersion* in a small quantity, or by the *vapor applied* merely for a limited period, a limb may be rendered *perfectly motionless and senseless*, and what may be an additional advantage, *fixed in any desired position.* He stated that he had immersed his finger in these fluids for about half an hour and an hour, and at the end of this period the finger was nearly powerless and insensible, and that it was forty-eight hours before the effects entirely disappeared, a sensation of heat and discomfort extending along the tract of the nerves to the axilla—that before operating on a difficult case for artificial pupil, he had applied for twenty minutes a small portion of the vapor of chloroform to the eye, by means of a small jar which accurately fitted the orbit, with the effect of rendering the parts nearly insensible. The first effects of these agents when locally applied is to produce redness, heat, and smarting, which subside, followed by swelling and redness of the integuments, which remain for some time. Mr. N. stated that he could completely paralyse any limb of frogs or toads by immersion or exposure to the vapor in about five minutes or less; and he mentioned, as a curious fact, that if the exposure to the influence were continued longer than was sufficient to produce a local effect, this influence extended to the corresponding limb of the other side: thus, for instance, if one hind leg became *too much* influenced, the other hind leg partook of the same effect—if the fore leg were too much effected, then the other fore leg became so likewise, and so on throughout the whole body—a result which Mr. Nunneley mentioned as strongly corroborative of his experiments with prussic acid, as detailed in the last volume of the Provincial Transactions, and strongly supporting the opinions of Dr. Marshall Hall on "reflex action." These views were illustrated by a series of interesting experiments before a highly respectable audience of medical men, on frogs and toads, in which, after immersion for a few minutes, the limbs became insensible, and were amputated in repeated portions without any symptoms of pain whatever.

The experiments which Mr. Nunneley performed before the meeting were perfectly successful and satisfactory; and if his views should prove to be correct, which we think very probable, they will give a new impulse to the use of anæsthetic agents, and enable the most cautious practitioner to use them without the danger that may attend their internal administration.

He stated that by this new mode of application to the hind legs of rabbits he had been enabled to amputate the toes without the least indication of feeling—that he was not prepared to state what was the best mode of applying it, or the exact quantity to be used, which obviously can only be determined by a very lengthy series of experiments on different animals, which he is at present zealously pursuing, his principal object being to communicate the important *physiological local effects* of anæsthetic agents generally, which we believe have not hitherto been announced.

